# Kidney segmentation in CT sequences using SKFCM and improved GrowCut algorithm

**DOI:** 10.1186/1752-0509-9-S5-S5

**Published:** 2015-09-01

**Authors:** Hong Song, Wei Kang, Qian Zhang, Shuliang Wang

**Affiliations:** 1 School of Software, Beijing Institute of Technology, Beijing, China; 2 School of Computer Science and Technology, Beijing Institute of Technology, Beijing, China

**Keywords:** Kidney segmentation, SKFCM, Improved GrowCut, Abdominal CT images

## Abstract

**Background:**

Organ segmentation is an important step in computer-aided diagnosis and pathology detection. Accurate kidney segmentation in abdominal computed tomography (CT) sequences is an essential and crucial task for surgical planning and navigation in kidney tumor ablation. However, kidney segmentation in CT is a substantially challenging work because the intensity values of kidney parenchyma are similar to those of adjacent structures.

**Results:**

In this paper, a coarse-to-fine method was applied to segment kidney from CT images, which consists two stages including rough segmentation and refined segmentation. The rough segmentation is based on a kernel fuzzy C-means algorithm with spatial information (SKFCM) algorithm and the refined segmentation is implemented with improved GrowCut (IGC) algorithm. The SKFCM algorithm introduces a kernel function and spatial constraint into fuzzy c-means clustering (FCM) algorithm. The IGC algorithm makes good use of the continuity of CT sequences in space which can automatically generate the seed labels and improve the efficiency of segmentation. The experimental results performed on the whole dataset of abdominal CT images have shown that the proposed method is accurate and efficient. The method provides a sensitivity of 95.46% with specificity of 99.82% and performs better than other related methods.

**Conclusions:**

Our method achieves high accuracy in kidney segmentation and considerably reduces the time and labor required for contour delineation. In addition, the method can be expanded to 3D segmentation directly without modification.

## Background

Image segmentation is one of most important issues in medical technology, which assists physicians in various aspects, such as analysis and diagnosis of different diseases, the study of anatomical structure, making treatment planning [1]. With the increase of CT images in the diagnosis and treatment of diseases, segmentation of human organs from CT images is a prerequisite step in the precise treatment planning. However, different tissues has different sizes and shapes across individuals and the gray scale similarity between kidney and its neighboring tissues, such as liver and spleen. Therefore kidney segmentation is a challenging work.

Many approaches of kidney segmentation have been developed over the recent years, including deformable model, clustering-based method, region growing and knowledge-based method. Tsagaan and Shimizu proposed a deformable model for automatic kidney segmentation which is represented by the grey level appearance of kidney and its statistical information of the shape [2,3]. Clustering method is a kind of unsupervised learning. So the segmentation methods based on it do not need training sample data, they form clusters of data by grouping pixels [4]. Lin developed an automatic method based on an adaptive region growing method to extract kidney within a region of interest (ROI). However this method mainly depended on the assumption of homogeneity of image intensity, so it is not suitable for the images that have large variation of intensity in the kidney region. Knowledge-based method makes use of the sample data for computing the extracting region, which needs computationally intensive work. Spiegel developed an algorithm based on 3D active shape model (ASM) [5]. Khalifa proposed a level-set method which combined a probabilistic shape prior and a novel stochastic function [6]. Region growing method is sensitive to the seed point location.

In the last decades, fuzzy segmentation methods, especially the fuzzy c-means algorithm (FCM) [7], have been widely used in the field of image segmentation [8]. There are many improved algorithms based on FCM. Zhang et al [9] proposed a kernel-based fuzzy c-means (KFCM) algorithm which has stronger noise immunity and clustering ability. In KFCM algorithm, a kernel-induced metric replaces the original Euclidean norm metric of FCM. In [10,11], FCM with spatial contextual information (FCM_S) is an effective image segmentation algorithm. Although the contextual information can raise its insensitivity to noise, it still lacks enough robustness to noise and outliers. To overcome these problems, S. Chen et al [12] proposed a novel KFCM algorithm which introduces a spatial constraint (SKFCM). The SKFCM algorithm was used to segment brain and tumor from MR images successfully [12-14].

Cellular automaton (CA) [15,16] is a nonlinear dynamic model which discrete in time and space and realizes a complex calculation by simple rules. The image processing methods based on cellular automata were used widely, including edge detection, segmentation and denoising. In 2006, Vladimir and Vadim [17] proposed the "GrowCut" algorithm which is an interactive segmentation method and solves pixel labeling task based on cellular automaton. Given some user-labeled points, the rest of the image is segmented automatically by a cellular automaton. The labeling process is iterative. Users can observe the segmentation evolution and guide the algorithm with human input where the segmentation is difficult to compute. The most common application of GrowCut algorithm is segmentation of brain tumors from MR images [17-19].

In this paper, a new coarse-to-fine method is proposed for kidney segmentation. It is a hierarchical segmentation framework combining SKFCM and IGC algorithm for the kidneys segmentation from abdominal CT images. In rough segmentation stage, SKFCM algorithm is better in segmenting images corrupted by noise than FCM algorithm. SKFCM adopts a kernel-induced metric in the data space to replace the original Euclidean norm metric in FCM, so it is a more robust clustering approach. The proposed IGC algorithm is used to refine the rough segmentation result. Due to the IGC algorithm makes good use of the continuity of CT sequences in space; it can generate both object and background seed labels automatically. The IGC algorithm can reduce a lot of interactive time and improve the efficiency of segmentation.

## Methods

**Figure 1:**
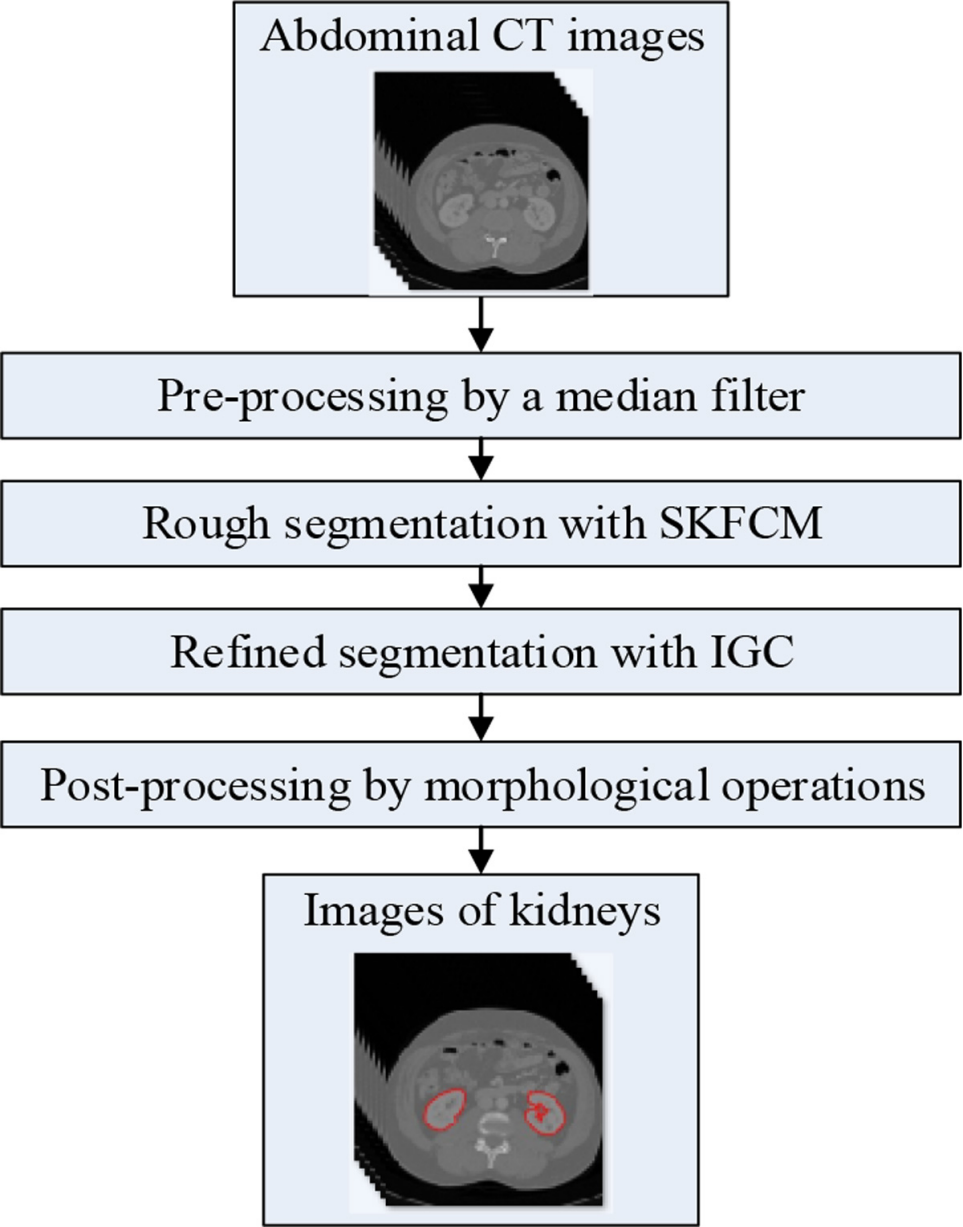
****The framework of the proposed kidney segmentation method****.

In this section, the proposed kidney segmentation method with a hierarchical strategy will be presented in detail. The hybrid method which incorporates SKFCM and IGC algorithm mainly consists of four steps. The framework of the proposed method is shown in Figure 1.

### A. Preprocessing

**Figure 2:**
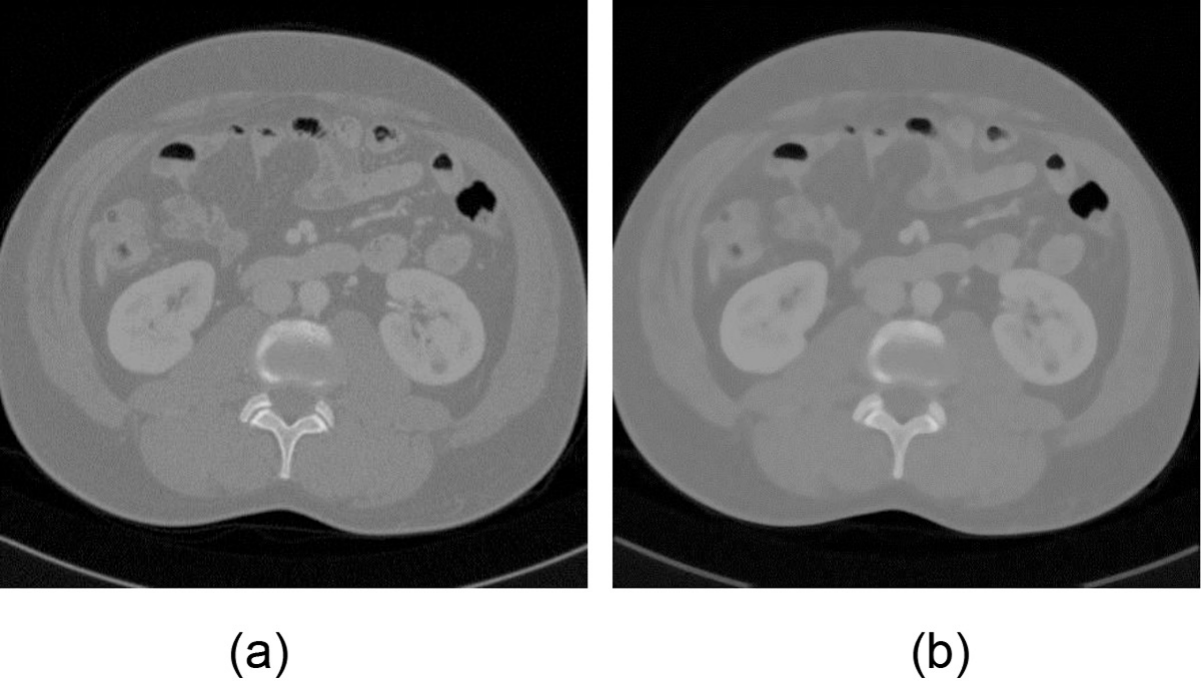
**Image preprocessing**. (a) the original image; (b) the denoised image by median filter.

CT image has inhomogeneity, noise which affect the continuity and accuracy of the images segmentation. Therefore, a 3 × 3 median filter is used to reduce the noise. The equation of median filter is defined as:

(1) $$\text{g}\left(\text{x},\text{y}\right)=\text{Median}\left(f_{s}\left(\text{x},\text{y}\right)\right)$$

where S represents the template window with a 3 × 3 surrounding neighborhood, $f_{s}\left(\text{x},\text{y}\right)$ represents the intensity value of each pixel in the S, $\text{Median}\left(f_{s}\left(\text{x},\text{y}\right)\right)$ represents the middle value of all the values of the pixels in the neighborhood. Instead of simply replacing the pixel value with the mean of neighboring pixel values, median filter replaces it with the median of those values. Figure 2(a) is the original image. Figure 2(b) is the denoised image by a median filter. It has a better denoising result while the edges are well preserved.

### B. Rough Segmentation with SKFCM

In this paper, SKFCM algorithm introduces a kernel function and spatial constraint into the FCM algorithm, which can reduce the effect of noise and improve the clustering ability. The objective function of SKFCM as follows,

(2) $$J_{m}=\overset{c}{\underset{i=1}{\sum }}\overset{N}{\underset{k=1}{\sum }}u_{ik}^{m}\left(1-K\left(x_{k},v_{i}\right)\right)+\alpha \overset{c}{\underset{i=1}{\sum }}\overset{N}{\underset{k=1}{\sum }}u_{ik}^{m}\left(1-K\left({\bar{x}}_{k},v_{i}\right)\right)$$

where c is the number of clusters of the dataset ${\left\{x_{k}\right\}}_{k=1}^{N}$, n is the number of pixels, m is a weighting exponent on each fuzzy membership and determines the amount of fuzziness of the resulting classification, ${\left\{v_{i}\right\}}_{i=1}^{c}$ are the centers and the array $\left\{u_{ik}\right\}\left(=U\right)$ represents a partition matrix, α in the second term controls the effect of the penalty, ${\bar{x}}_{k}$ is the mean of neighboring pixel values of $x_{k}$, $\text{K}\left(\cdot ,\cdot \right)$ is the Gaussian kernel function. The objective function in (2) is minimized using the alternate iterations of the fuzzy partition matrix (3) and the centroids of clusters (4).

(3) $$u_{ik}=\frac{{\left(\left(1-K\left(x_{k},v_{i}\right)\right)+\alpha \left(1-K\left({\bar{x}}_{k},v_{i}\right)\right)\right)}^{\frac{-1}{\left(m-1\right)}}}{\sum_{j=1}^{c}{\left(\left(1-K\left(x_{k},v_{i}\right)\right)+\alpha \left(1-K\left({\bar{x}}_{k},v_{i}\right)\right)\right)}^{\frac{-1}{\left(m-1\right)}}}$$

(4) $$v_{i}=\frac{\sum_{k=1}^{n}u_{ik}^{m}\left(K\left(x_{k},v_{i}\right)x_{k}+\alpha K\left({\bar{x}}_{k},v_{i}\right){\bar{x}}_{k}\right)}{\sum_{k=1}^{n}u_{ik}^{m}\left(K\left(x_{k},v_{i}\right)+\alpha K\left({\bar{x}}_{k},v_{i}\right)\right)}$$

The above algorithm can be summarized in the following steps.

Step 1: Fix the number c of these centroids and select initial class centroids and set ε>0 to a very small value.

Step 2: Compute the mean filtered image.

Step 3: Update the partition matrix using (3).

Step 4: Update the centroids using (4)

Repeat steps 3-4 until the following termination criterion (5) is satisfied:

$$V_{new}-V_{old}<\varepsilon$$

The purpose of this subsection is to get the rough contour of kidney in the CT images. Owing to the gray scales of kidney is similar to its neighboring tissues, it is important to identify which part belongs to the kidney. To solve this problem, a slice which has the largest contour in the whole dataset is cropped by a rectangle manually. The rectangle must enclose the kidney and its size should be as small as possible, so that it can increase the processing speed and the segmentation accuracy. Other slices are automatically cropped as described in the following. The optimal cluster number is 4 which is determined by experiments. The rough segmentation includes six steps.

Step 1: The cropped image is the input of SKFCM algorithm, and then each pixel in the cropped region is clustered into different clusters.

Step 2: The number of pixels in each cluster is calculated and the cluster which contains maximum pixels is extracted.

Step 3: The largest connected region is extracted to be the candidate kidney region.

Step 4: There are some holes inside the kidney because some vessels are rejected in the processing of fuzzy clustering. Therefore this step is to fill holes.

Step 5: The kidney contour is smoothed by morphological operations.

Step 6: Through the above steps, the mask of candidate kidney region is gotten. In order to realize the continuous segmentation, the minimum bounding rectangle (MBR) of the mask is calculated. Then the MBR is extended about 10 pixels so that we can get a new rectangle. This new rectangle is used to crop the next slice of CT sequeces.

**Figure 3:**
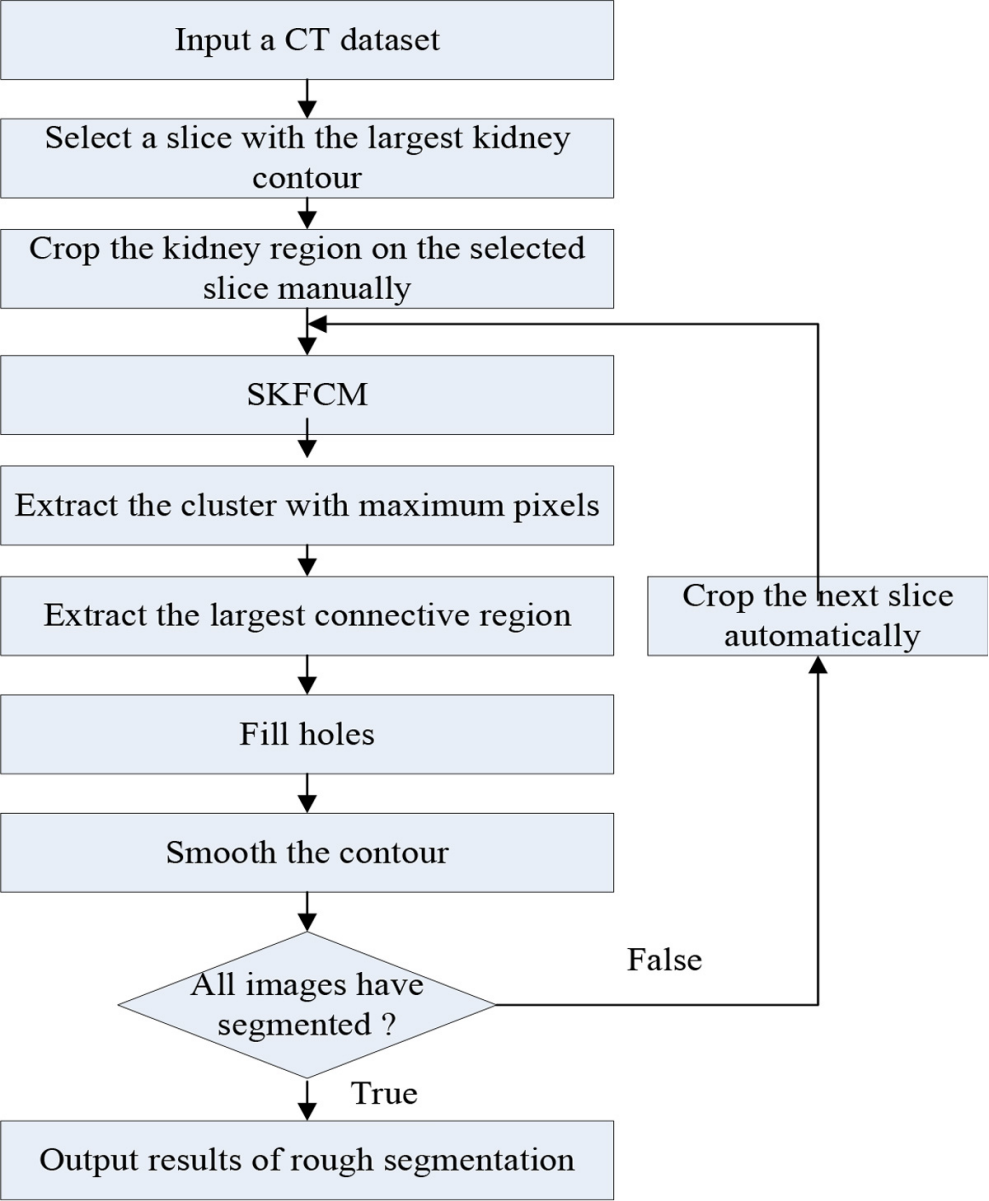
**The procedure of rough segmentation**.

Continuous rough kidney segmentation on CT images can be achieved by repeating steps 1-6. The procedure of rough segmentation is shown in Figure 3.

**Figure 4:**
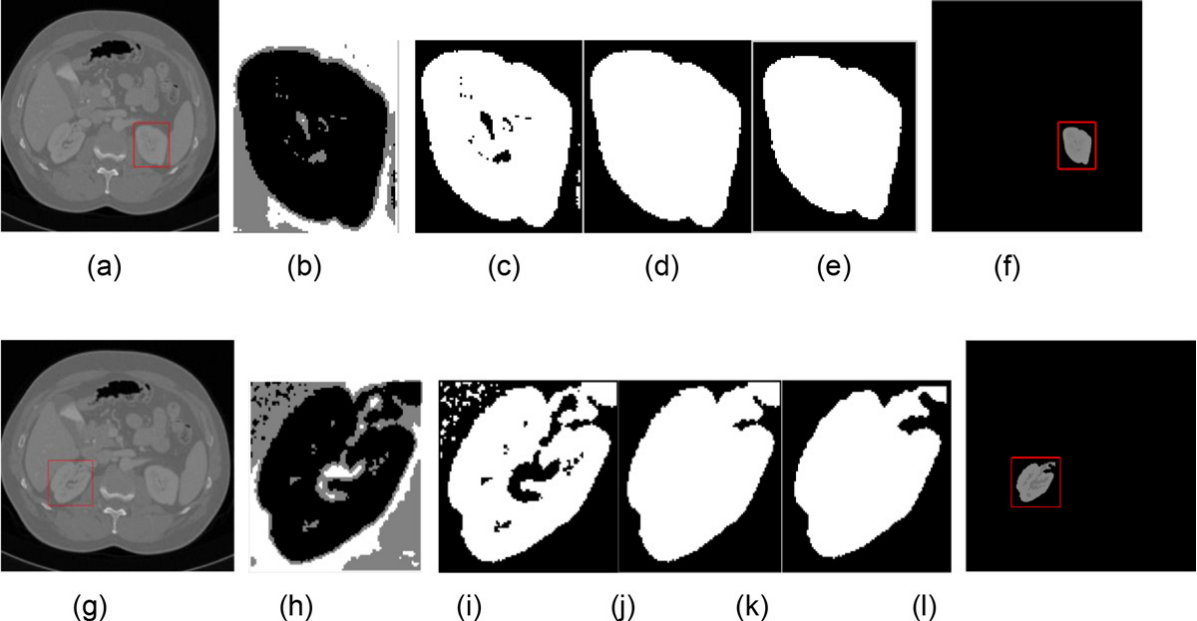
**Examples of rough segmentation**. (a) and (g) are original images with cropping rectangle; (b) and (d) are clustering results; (c) and (i) are the extracted clusters with maximum pixels; (d) and (g) are the largest connective regions with filled holes; (e) and (k) are the smooth results; (f) and (l) are the rough segmentation results and the red rectangles are used to crop next slice.

**Figure 5:**
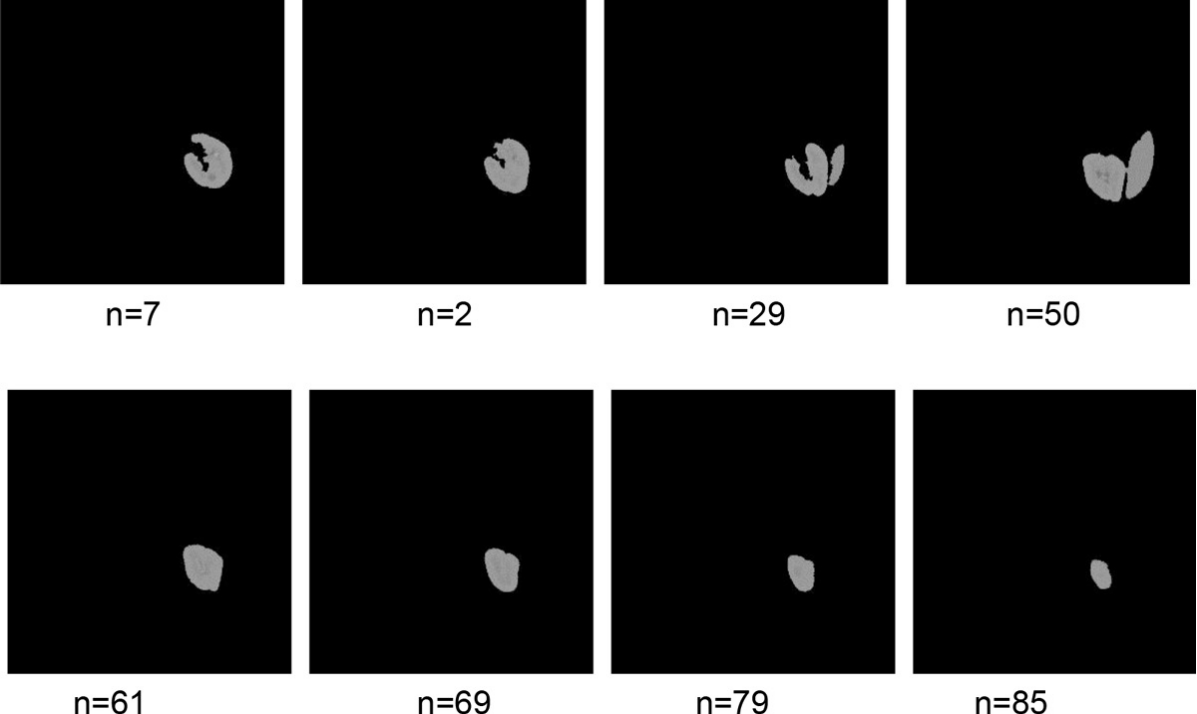
**Some results of the rough segmentation**.

Figure 4 shows some intermediate results of rough segmentation. Figure 4(a) and 4(g) are original images, and the red rectangles are used to crop these original images. Figure 4(b) and 4(h) are clustering results. Figure 4(c) and 4(i) are the clusters which contain maximum pixels. Figure 4(d) and 4(j) are the largest connective regions with filled holes. Figure 4(e) and 4(k) are the candidate kidney with smooth contour. Figure 4(f) and 4(l) are the final results of SKFCM. The red rectangle is generated automatically and used to crop the next slice of CT images. As shown in the figures, Figure 4(f) is a better segmentation result than Figure 4(l). The former does not need refined segmentation, but the later need further segmentation by IGC algorithm. In the whole datasets, there are about half of images do not need refined segmentation after rough segmentation. More results of rough segmentation were shown in Figure 5. The parameter *n *is the slice number in the whole datasets.

### C. Refined Segmentation with IGC Algorithm

#### 1) The traditional GrowCut

GrowCut algorithm is an interactive segmentation method and solves pixel labeling task based on cellular automaton.

A cellular automaton (CA) is defined as a triplet $\text{A}=\left(\text{S},\text{N},\delta \right)$, where S is a set of non-empty state, N is the neighborhood system and $\delta :{\text{S}}^{\text{N}}\to \text{S}$ defines the state transition rule of cells at time t+1 based on the states of neighbor cells at time t. The Moore von (8-connected) and Neumann (4-connected) neighborhoods are two commonly used neighbor systems. The state of each cell is also a tri-plet ${\text{S}}_{\text{p}}=\left({\text{l}}_{\text{p}},\theta_{\text{p}},{\vec{\text{C}}}_{\text{p}}\right)$, where ${\text{l}}_{\text{p}}$ is the label of this cell, $\theta_{\text{p}}$ is the strength of this cell which ranges from 0 to 1, and ${\vec{\text{C}}}_{\text{p}}$ is the feature vector that its value is image intensity.

A two-dimensional medical image (P) is a matrix of m×n pixels, and it is also treated as a cellular automaton with a special state in GrowCut algorithm. Each pixel (p) in the image is a "cell" with a certain type, and it may be background, foreground or undefined. The initial state for ∀p∈Pis set to

(6) $${\text{l}}_{\text{p}}=0;\theta_{\text{p}}=0;{\vec{\text{C}}}_{\text{p}}={\text{I}}_{\text{p}}$$

where ${\text{l}}_{\text{p}}$ is the intensity value of each pixel. As the segmentation algorithm proceeds, all pixels in this image are assigned to one of possible labels.

Before starting the segmentation, user should input an initial label matrix manually. The label matrix has a same size with the original image. In the label matrix, there are two kinds of marked points, one is foreground seed point whose label is ${\text{l}}_{\text{p}}=1$, and the other is background seed point whose label is ${\text{l}}_{\text{p}}=-1$. The original strength of these two kinds of marked points is $\theta_{\text{p}}=1$. Apart from these two kinds of marked points, the label of the remainder of points is ${\text{l}}_{\text{p}}=0$. After all the initial operations have been done, the iteration segmentation runs until the label matrix does not change. Finally, the label value of object region is 1 and the label value of background is -1. The iterative process of labels ${\text{l}}_{\text{p}}$ and strength $\theta_{\text{p}}$ at time t+1 is shown as follows,

      State transition of CA

      // For each cell...

        for ∀p∈P

      // Copy the previous state

        ${\text{l}}_{\text{p}}^{\text{t}+1}={\text{l}}_{\text{p}}^{\text{t}}$;

        $\theta_{\text{p}}^{\text{t}+1}=\theta_{\text{p}}^{\text{t}}$;

        // Neighbors try to attack current cell

    for ∀q∈N(p)

            if $\text{g}\left(||{\vec{\text{C}}}_{\text{p}}-{\vec{\text{C}}}_{\text{q}}|{|}_{2}\right)\cdot \theta_{\text{q}}^{\text{t}}>\theta_{\text{p}}^{\text{t}}$

            ${\text{l}}_{\text{p}}^{\text{t}+1}={\text{l}}_{\text{q}}^{\text{t}}$;

            $\theta_{\text{p}}^{\text{t}+1}=\text{g}\left(\text{||}{\vec{\text{C}}}_{\text{p}}-{\vec{\text{C}}}_{\text{q}}||{}_{2}\right)\cdot \theta_{\text{q}}^{\text{t}}$;

        end if

    end for

end for

In [18], g(x) is defined as:

(7) $$\text{g}\left(\text{x}\right)=1-\frac{\text{x}}{\text{max}{||\vec{\text{C}}||}_{2}}$$

#### 2) The Improved GrowCut

Although GrowCut algorithm is simple and precise, it is an interactive segmentation algorithm. For some complex images, just once interactive operation cannot achieve satisfactory results, and both foreground seed points and background seed points also should be selected carefully. In order to avoid multi-interaction, we propose an improved GrowCut algorithm which can generate seed labels automatically.

**Figure 6:**
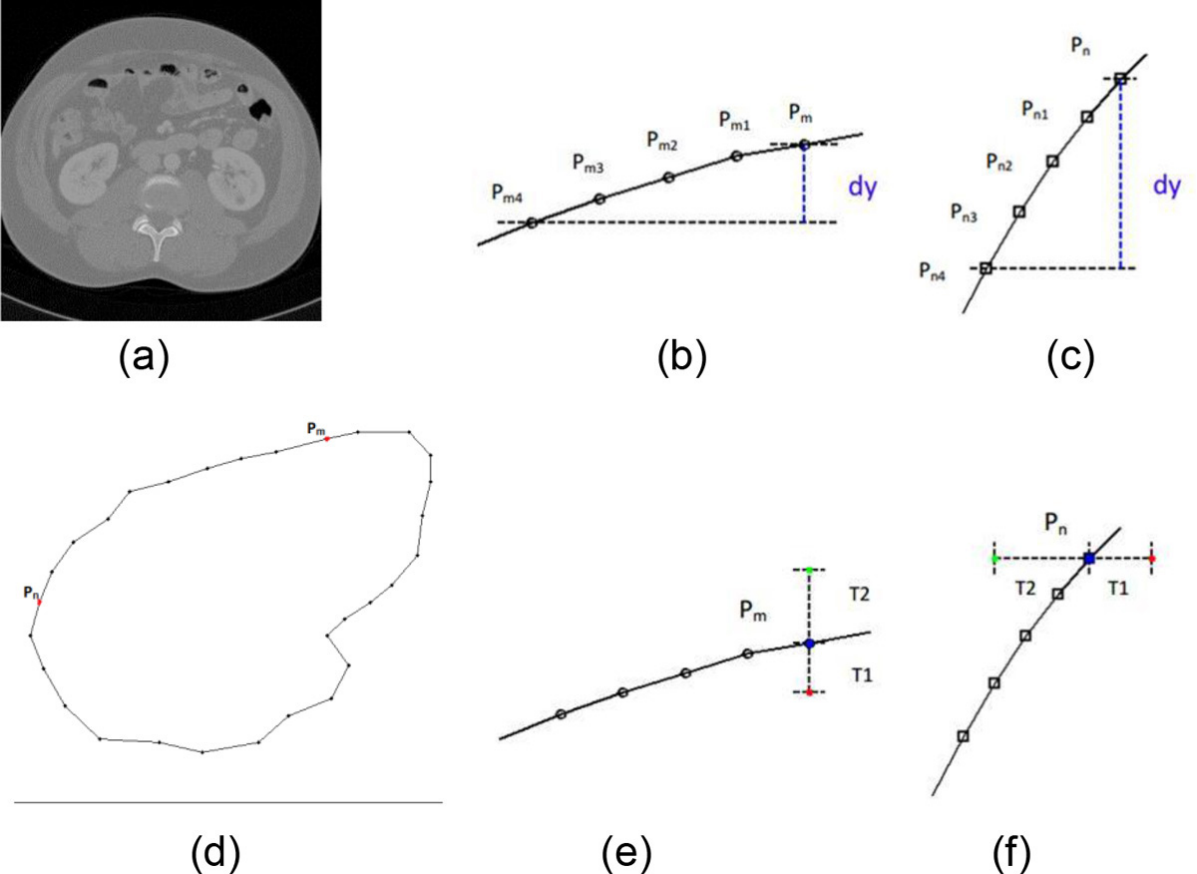
**The example of generating the seed template automatically**. (a) an original image of template; (b) altitude difference of point P_m_; (c) altitude difference of point P_n_; (d) the contour of template; (e)the foreground seed point (red) and background seed point (green) of point P_m_; (f) the foreground seed point (red) and background seed point (green) of point P_n_.

In this subsection, the rough segmentation results are continuous in space series and about half of them do not need refined segmentation. Therefore, the initial label matrix can be generated automatically using the result of rough segmentation (denoted as "a seed template image"). The seed template image must meet two conditions. One is that it does not need refined segmentation, and the other is that it is adjacent to the slice which needs refined segmentation. The process of generating both foreground and background seed points is described in Figure 6. Figure 6(a) is an original kidney CT image and Figure 6(d) is the contour of the kidney. Due to the spatial continuity of CT images, Figure 6(d) can be used to generate the initial label matrix for the segmentation of next slice. There are four steps to generate the initial label matrix automatically.

Firstly, we should get the edge points of kidney contour.

Secondly, calculate the altitude difference between two edge points. Figure 6(b) and 6(c) show the altitude difference (d_y_) at point P_m _and P_n _respectively.

Thirdly, there are three threshold values T_h_, T_1_, T_2 _to control the process of generating seed points. The seed points will be located in the vertical direction if d_y _is less than T_h_, otherwise they will be located in the horizontal direction. Taking Figure 6(e) as an example, the foreground seed point is located at the bottom of P_m _and the background seed point is located at the top of P_m _, because d_y _is less than T_h_. In Figure 6(f), the foreground seed point is located at the right of P_n _and the background seed point is located at the left of P_n_, because d_y _is greater than T_h_. All foreground seed points are located inside of the kidney contour and the distance between them and edge points are T_1 _pixel. All background seed points are located outside of the kidney contour and the distance between them and edge points are T_2 _pixel. In Figure 6(e) and 6(f), the red points are denoted as foreground seed points and the green points are denoted as background seed points.

Finally, according to step 2 and 3, we can get both foreground and background seed points of each edge point

**Figure 7:**
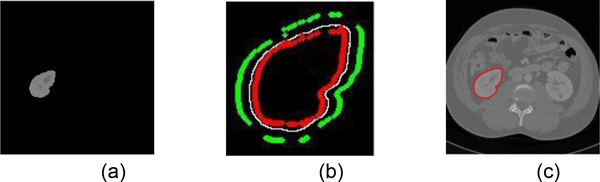
**The refined kidney segmentation based on IGC algorithm**. (a) the seed template image; (b) the seed label image which is generated automatically by IGC, the red points are foreground seed points and the green points are background seed points; (c) result by IGC algorithm.

**Figure 8:**
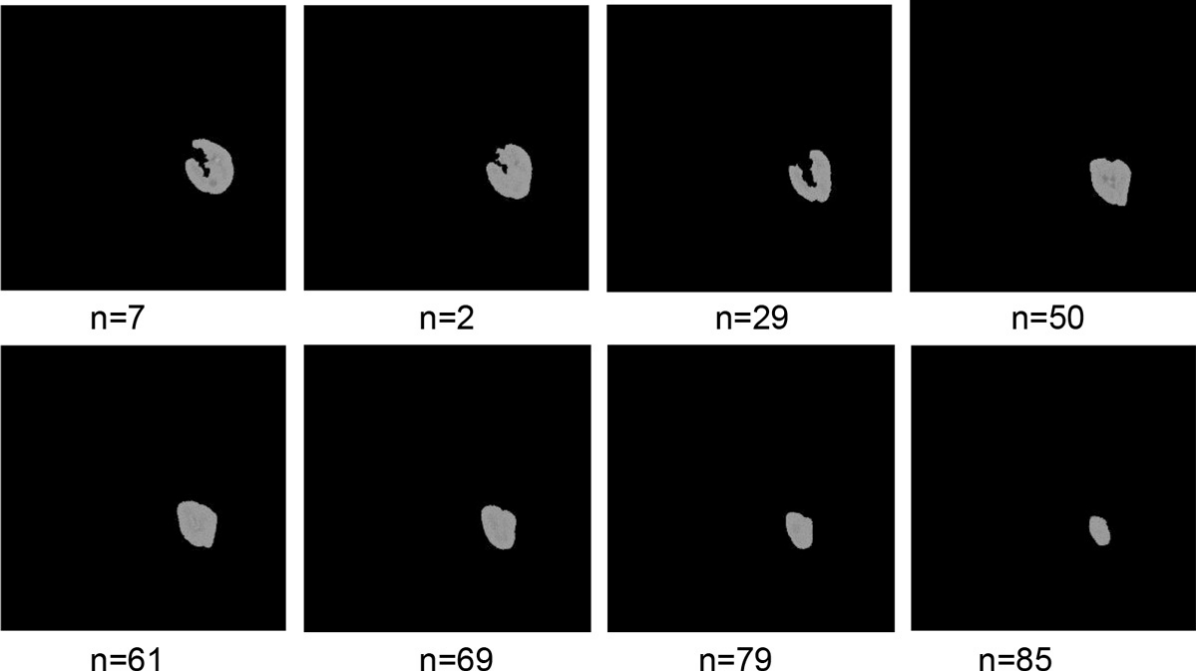
**Some results of the refined segmentation**.

Figure 7 shows the refined segmentation result by IGC algorithm. Figure 7(a) is a seed template image. Figure 7(b) is the seed label image which is generated automatically base on IGC algorithm. The red points are foreground seed points and the green points are background seed points. The value of red point is 1, the value of green point is -1, and the remainders are 0. The final result of IGC is shown in Figure 7(c). More refined segmentation results are shown in Figure 8. They have the same slice number with the rough segmentation results in Figure 5.

### D. Post-processing

Some segmentation results of IGC and SKFCM have rough boundaries. To achieve a smoother contour of kidney, a post-processing method based on morphological operations is needed. The most common morphological operations are dilation and erosion.

## Results and evaluation

The segmentation experiments and performance evaluation were carried on three groups of abdominal CT images. The parameters of abdominal CT images for scanning were 120.0 KV and 297.0 mA. The pixel spacing was 0.683594 mm, the slice thickness was 1.0 mm and the spacing between slices was 0.5 mm. The number of slices ranged from 217 to 320. Each slice of these three datasets had a spatial resolution of 512 × 512 pixels. Both SKFCM and IGC algorithm were implemented on MATLAB R2013b. All experiments were implemented on the computer with Pentium Dual - Core CPU (2.80GHz) and 2GB memory.

In order to prove the advantages of IGC algorithm, the results which are segmented by the proposed IGC algorithm are compared with those gotten by traditional GrowCut algorithm (TGC). For quantitative evaluation of the IGC algorithm, there are four evaluation criterions: accuracy, overlap, the number of interactions (NOI) and the time of generating seed points by manual method or computer algorithm method (TOGSP). Accuracy is a common criterion that it is used to evaluate performance of segmentation methods widely. Overlap shows the degree of overlap between segmentation results by computer algorithm and manual segmentation results. The closer overlap is to 1, the better segmentation result will be. The accuracy and overlap are defined as follows:

(8) $$\text{accuracy}=\frac{TP+TN}{TP+FP+TN+FN}$$

(9) $$\text{overlap}=\frac{TP}{FN+TP+FP}$$

where TP denotes the number of true positive pixels which are correctly classified as kidney when they are actually kidney. TN denotes the number of true negative pixels which are correctly classified as non-kidney when they are actually non-kidney. FP denotes the number of false positive pixels which are correctly classified as kidney when they are kidney. FN denotes the number of false negative pixels which are correctly classified as non-kidney when they are kidney. The definition of TP, TN, FP and FN is shown in Table 1.

**Table 1:** The definition of TP FP FN and TN.

		Result by manual segmentation	
		
		Positive	Negative
Result by segmentation algorithm	Positive	TP	FP
	
	Negative	FN	TN

**Figure 9:**
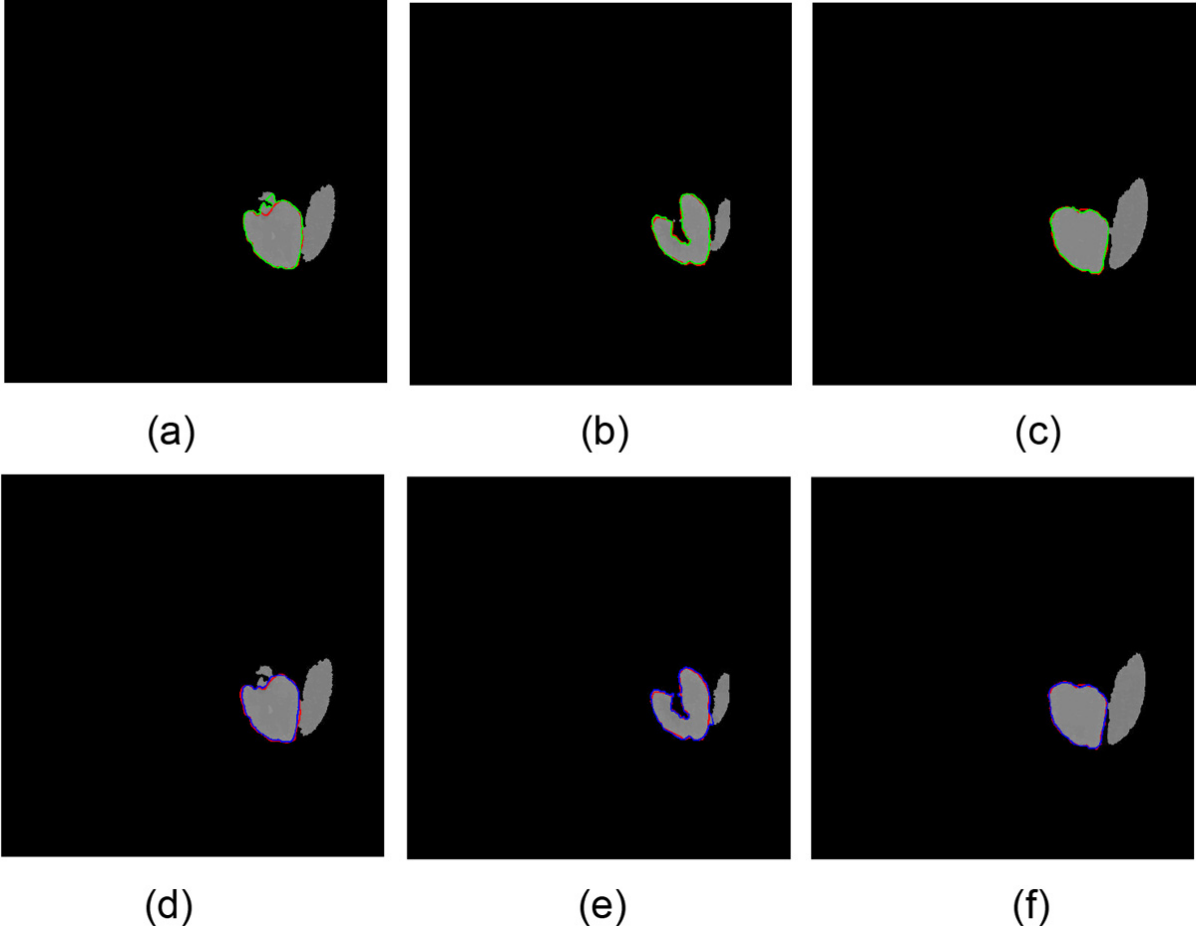
**Results of refined segmentation by different algorithms**. (a)(b)(c) show the refined segmentation results of TGC algorithm; (d)(e)(f) show results of IGC algorithm; the red line denotes the manual segmentation result; the green line denotes the result of TGC algorithm; the blue line denotes the result of IGC algorithm.

In order to compare the performance of TGC and IGC, we choose three slices from rough segmentation results to implement refined segmentation. The evaluation value of different methods is shown in Table 2. Compared with the TGC algorithm which is a semi-automatic method and needs manually select foreground seed points and background seed points subjectively, the proposed IGC algorithm is more accurate and has a high overlap. Above all, the proposed IGC can reduce a lot of interactive time. The refined results by TGC algorithm need at least once interaction and the average time of once interaction is more than ten seconds. Therefore, IGC algorithm shows more efficient than TGC algorithm. In Figure 9, the top row is the segmentation results of TGC algorithm, whose contour color is green, and the bottom row is the segmentation results of IGC, whose contour is blue. The red contour is the manual segmentation result, which is the ground truth data. We can see that the contour of kidney extracted by IGC is closer to the real contour than TGC. Through the above quantitative and qualitative analysis, IGC algorithm is better than TGC in image segmentation.

**Table 2:** The evaluation of different algorithms.

	Methods	Accuracy (%)	Overlap (%)	NOI	TOGSP (s)
Data 1	TGC	99.69	86.61	1	29.14
	IGC	99.64	85.11	0	0.50

Data 2	TGC	99.59	80.71	2	25.23
	IGC	99.62	82.57	0	0.51

Data 3	TGC	99.69	86.59	1	38.28
	IGC	99.72	88.08	0	0.50

The proposed method was also compared with other kidney segmentation methods quantitatively. We adopted accuracy, sensitivity and specificity as the criterions. Sensitivity means how many kidney tissues are accepted in the outcome compared with ground truth. Specificity shows how many non-kidney tissues are rejected in the outcome. The definition of sensitivity and specificity are:

(10) $$\text{sensitivity}=\frac{TP+TN}{TP+FP+TN+FN}$$

(11) $$\text{specificity}=\frac{TP}{FN+TP+FP}$$

We use three groups of abdominal CT data from three different patients to test the performance of our method and compare with other methods. Average results of these criterions achieved by our method and others are summarized in Table 3. In [20] Hu et al. proposed a method of kidney segmentation which is based on statistical conditional random fields framework. In [21] the method of kidney segmentation proposed by Belgherbi et al. is based on the anatomical information and mathematical morphology tools used in the image processing filed. As we can see from Table 3 the accuracy, sensitivity and specificity of our methods is higher than the methods of ref.20 and ref.21. The higher sensitivity and specificity illustrate that our method can achieve a higher recognition rate of kidney area and non-kidney area. The segmentation results of our method can meet the needs of image analysis and clinical research.

**Table 3:** The average results of metrics achieved by our method and some other methods.

Method	Accuracy (%)	Sensitivity (%)	Specificity
Hu [20]	Less than 99.0	92.50	99.50
Belgherbi [21]	99.00	95.00	99.00
Our method	99.66	95.46	99.82

## Conclusion

In this paper, we proposed a new coarse-to-fine method that combines SKFCM and the improved GrowCut algorithm to extract the kidneys for the abdominal CT images. The method was tested on the whole dataset of abdominal CT images. Experimental results have been shown visually and achieve reasonable consistency. The performance evaluation of segmentation results demonstrates that our kidney segmentation method is accurate and efficient. There are two key contributions. First, SKFCM algorithm is used to implement rough kidney segmentation successfully due to its strong clustering ability and robust noise immunity. Second, the traditional GrowCut algorithm has been improved. The improved GrowCut algorithm can generate seed labels automatically instead of inputting seed labels by users, so that it can reduce interactive time and improve the efficiency of segmentation. The segmentation results of our method can be used to diagnose the kidney diseases and make treatment planning. They are also the foundation of 3D visualization.

## Competing interests

The authors declare that they have no competing interests.

## Authors' contributions

Hong Song proposed the algorithm and revised the manuscript. Wei Kang did the experiments and analyzed the data. Qian Zhang implemented the algorithm and wrote the manuscript. Shuliang Wang revised the manuscript. All authors read and approved the final manuscript.

## Publication funding from grants

The publication charges for this article were funded by the National Natural Science Foundation of China grant 61240010.
